# Cardiac function in children with congenital diaphragmatic hernia: cardiac strain at birth and at 2–5 weeks of age

**DOI:** 10.3389/fped.2025.1598695

**Published:** 2025-07-17

**Authors:** Katarina Övermo Tydén, Kerstin Magnusson, Carmen Mesas Burgos, Baldvin Jonsson, Felicia Nordenstam

**Affiliations:** ^1^ Department of Women’s and Children’s Health, Karolinska Institutet, Stockholm, Sweden; ^2^ Department of Pediatric Cardiology, Karolinska University Hospital, Stockholm, Sweden; ^3^ Department of Pediatric Surgery, Karolinska University Hospital, Stockholm, Sweden; ^4^ ECMO Centre, Karolinska University Hospital, Stockholm, Sweden

**Keywords:** CDH, congenital diaphragmatic hernia, cardiac function, neonates, strain

## Abstract

**Introduction:**

Neonates with congenital diaphragmatic hernia (CDH) often present with pulmonary hypertension and various forms of cardiac dysfunction, affecting right or left ventricle or both. Although pulmonary hypertension typically improves over time, some children experience long term pulmonary hypertension. There is limited understanding of cardiac function recovery. The authors hypothesized that cardiac function in the CDH population would remain impaired during the first 2–5 weeks of life compared with in a control group.

**Method:**

This prospective observational cohort study included 40 newborns with CDH and 40 controls born in 2021–2024 and cared for at Karolinska University Hospital, Stockholm, Sweden. The first study echocardiograms were performed between 6 and 24 h of age. Subsequently, 23 of the 40 CDH cases were also examined at 2–5 weeks of age. Left ventricular longitudinal strain (LV LS), right ventricle free wall strain (RVFWS), left atrial reservoir strain (LASr), right atrial reservoir strain (RASr), and conventional echo parameters were analyzed.

**Results:**

Newborns with CDH had reduced cardiac function measured with LV LS [mean −14.7% (standard deviation 4.6) vs. −18.8% (2.7), *p* < 0.001], RVFWS [−13.9% (4.2) vs. −22.4% (4.8) *p* < 0.001], LASr [22.8% (9.3) vs. 33.9% (8.2), *p* < 0.001], and RASr [21.8% (6.9) vs. 37.6% (9.2), *p* < 0.001], compared with controls. Cardiac strain had improved for all subjects at the age of 2–5 weeks. However, the cases with CDH still had lower strain than the controls.

**Conclusion:**

Cardiac function measured as strain was reduced in newborns with CDH compared with in a control group. Although their cardiac function improved over time, it remained lower compared to the control group at 2–5 weeks of age.

## Introduction

The care of congenital diaphragmatic hernia (CDH) has improved over the last decades, resulting in an increased survival rate. However, the most severe cases still have a mortality rate around 50% (1, 2). Several studies have shown that the degree of cardiac dysfunction is a key risk factor (3–6). Emerging evidence suggests that cardiac dysfunction in CDH can be present in different phenotypes; including pulmonary hypertension and right ventricle (RV) dysfunction, left ventricle (LV) dysfunction and biventricular dysfunction, with pulmonary hypertension and right ventricle dysfunction being the center of attention (7). However, left ventricle dysfunction is common in CDH and may complicate the course of intensive care (4, 8). The complexity of the cardiac dysfunction urges the need for an early echocardiogram assessment that guides towards the most suitable choice of care and treatment (9). Strain, a measurement of myocardial deformation, has emerged as a novel echocardiographic assessment of cardiac function (10). Left ventricular longitudinal strain (LV LS), right ventricle free wall strain (RVFWS), and left atrial reservoir strain (LASr) have all been found to correlate with length of stay (LOS) in a pediatric intensive care unit (PICU), risk of needing extra corporeal membrane oxygenation (ECMO), and mortality in newborns with CDH (11–13). Right and left atrial strain (RAS; LAS) encompass all three phases of atrial function: reservoir (r), conduit (cd), and contractile (ct). Right atrial reservoir strain (RASr) has shown potential as a prognostic marker in adult patients with pulmonary hypertension and impaired heart function (14). However, the clinical use of RAS is ambiguous (15). Pediatric studies have identified RAS as an indicator of right ventricle diastolic dysfunction and a predictor of pulmonary hypertension (16, 17). To our knowledge, there are no studies in the CDH population regarding RAS. As the survival rate of children born with CDH has improved, more of the severe CDH cases survive and long-term morbidity has increased (18). Little is known about recovery of cardiac function in CDH survivors, as there are few studies and there is a lack of structured long-term follow-up programs (19, 20). However, it seems that being born with CDH is a risk factor for later cardiac morbidity (20, 21).

To support cardiac function, a phosphodiesterase 3 inhibitor like milrinone can be used in the intensive care setting. Milrinone increases cardiac contractility and decreases systemic and pulmonary vascular resistance. The use of milrinone in neonatal and pediatric intensive care settings has increased, also in the management of the newborn CDH population (22, 23). However, only a few studies regarding CDH and the use of milrinone in the PICU have been published – and those show conflicting results (24–26). In Sweden, the care of CDH has been centralized since 2018, with Karolinska University Hospital being one of two national referral centers, facilitating studies of this group.

We hypothesized that cardiac function measured as strain would be impaired at birth and remain impaired at 2–5 weeks of age in the cohort of neonates with CDH compared with a control group. We further hypothesized that RAS would be associated with RV strain and conventional indirect measurements of pulmonary hypertension. Additionally, we hypothesized that milrinone treatment would shorten LOS in the PICU, reduce the duration of invasive mechanical ventilation (IMV), and facilitate cardiac function recovery.

## Materials and methods

### Study design

This was a prospective observational cohort study including a control group. All medical data were retrieved from medical records at the Karolinska University Hospital. Echocardiographic examinations were first performed at between 6 and 24 h after birth in a cohort of neonates with CDH (*n* = 40) and an age-matched control group (*n* = 40). Examinations were also performed at a second timepoint, at 2–5 weeks of age, in a subgroup of the CDH group (*n* = 24) and the control group (*n* = 23). At Karolinska University Hospital, all children born at term in need of mechanical support, including nasal continuous positive airway pressure, are treated in the PICU. This study was designed and reported in accordance with the STROBE (Strengthening the Reporting of Observational Studies in Epidemiology) guidelines to ensure clarity and transparency (27).

### Study population

This study included a cohort of 40 neonates born with CDH between January 2021 and March 2024 and treated at Karolinska University Hospital, Stockholm, Sweden. The control group consisted of 40 newborns, either with surgical conditions not related to the heart or healthy newborns, cared for in the maternity ward. All children included in the control group were cared for at Karolinska University Hospital. All participants were enrolled after verbal and written information was provided to the caregivers and written consent was obtained. The hernia severity was assessed prenatally based on observed-to-expected lung-to-head ratio and postnatally based on the need for patch and defect size (28). A few children born with CDH needed tracheostomy and long-term IMV, due to pulmonary hypoplasia and complications due to mechanical ventilation including tracheal stenosis rather than cardiac dysfunction. Therefore, in this study, neonates with more than 30 days' duration of IMV were excluded from certain echocardiographic measurements. Major congenital heart defects were defined as severe heart defects present at birth that significantly affect heart function and require surgery within the first year. These were also an exclusion criterion.

### Standardized hemodynamic considerations

During the study we followed specific hemodynamic guidelines for managing neonates with CDH at our unit in Karolinska University Hospital. If pre- and post-ductal saturations differed by more than 10%, and the echocardiogram revealed either supra-systemic or severe pulmonary hypertension associated with right ventricular dysfunction, treatment with inhaled nitric oxide (iNO), often in combination with milrinone were considered. ECMO was considered based on our unit's criteria, which included the following: pre-ductal saturation < 85%, oxygenation index > 40, rising pCO2 and lactate levels, acidosis, and hypotension.

Regarding inotropic therapy, the first-line choice was noradrenaline. Milrinone was almost always used in conjunction with noradrenaline to address the tendency for hypotension. In cases requiring more than one inotropic agent, vasopressin, adrenaline, or both were utilized.

### Echocardiography

Eighty neonates had an echocardiogram performed within 24 h of birth. Twenty-four of the cases and 23 controls enrolled had a follow-up echocardiogram at the age of 2–5 weeks. All these echocardiographic examinations were performed in accordance with a specific study protocol with optimized frame rate based on echocardiographic conditions. Echocardiograms were performed using Philips Epiq Elite 7.0.5 (Koninklijke Philips N.V.) and a S9-2 or a S -12 transducer. All examinations were performed by three experienced sonographers or one experienced pediatric cardiologist (KÖT)*.* All data from the examinations were analyzed by one experienced pediatric cardiologist (KÖT) and one experienced sonographer (KM)*.* Conventional echocardiographic measures analyzed were left ventricle end diastolic dimension (LVED) and shortening fraction (SF), measured with M mode in parasternal length axis and presented with Z scores, as described by Pettersen et al. (29). Tricuspid annular plane systolic excursion (TAPSE), pulmonary artery acceleration time (PAAT), eccentricity index (EI), and right ventricle to left ventricle (RV/LV) ratio were all measured in accordance with guidelines (30). Septal deviation was graded in parasternal short axis view, and categorized as round, flattened, or D-shaped. The direction of blood flow through the patent foramen ovale (PFO) and the patent ductus arteriosus (PDA), respectively, was categorized as left to right, bidirectional, or right to left. For myocardial strain, the speckle tracking analysis model was used. In accordance with the EACVi/ASA Industry Task Force guidelines for standardizing of deformation imaging, echocardiograms with poor quality inadequate for strain measurement were excluded (31). The endocardial border was tracked automatically, using TomTec version TTA2 (TOMTEC Imaging Systems GmbH, Unterschleissheim, Germany). The tracking was manually adjusted when needed. LAS and RAS were measured in apical four-chamber view, with optimization of the image for each cardiac atrium to avoid foreshortening. Pulmonary veins and/or the appendage orifices were excluded (32). Both LAS and RAS are presented as peak strain or reservoir, conduit, and contractile strain. Left ventricular strain was measured as peak left ventricular longitudinal strain (LV LS) from an apical four-chamber view (33–35). Right ventricular strain was measured from right ventricle-focused apical view and presented as right ventricular free wall strain (RVFWS) (35, 36).

### Statistics

Categorical data are expressed as frequencies or proportions. Continuous data are presented as mean [standard deviation (SD)] or median [interquartile range (IQR)]. Normally distributed data were analyzed with independent sample Student's *t*-test to assess differences between groups whereas non-normally distributed data were analyzed with the Mann–Whitney *U*-test. For comparisons involving more than two groups, the Kruskal Wallis test was applied. For nominal categorical data, the chi-squared test or Fisher's exact test was used. Spearman's rank correlation coefficient was calculated to assess linear relationships between variables. Uni- and multivariate models were used and compared using forward selection. A *p*-value < 0.05 was considered statistically significant, and 95% confidence intervals were reported. Statistical analyses were performed using SPSS software (version 29; IBM).

### Intra-observer and inter-observer variability test

Ten randomly selected echocardiograms, blinded to the observer, were measured twice by the same observer with an interval between the measurements of >8 weeks. Fifteen randomly selected exams were measured by two different observers (KÖT and KM). Intra- and inter-rater variability was calculated using SPSS intraclass correlation, 2-way mixed effects model.

### Ethics

This study was approved by the Swedish ethical review board, dnr 2020-03543.

## Results

### Study cohort

**Figure 1:**
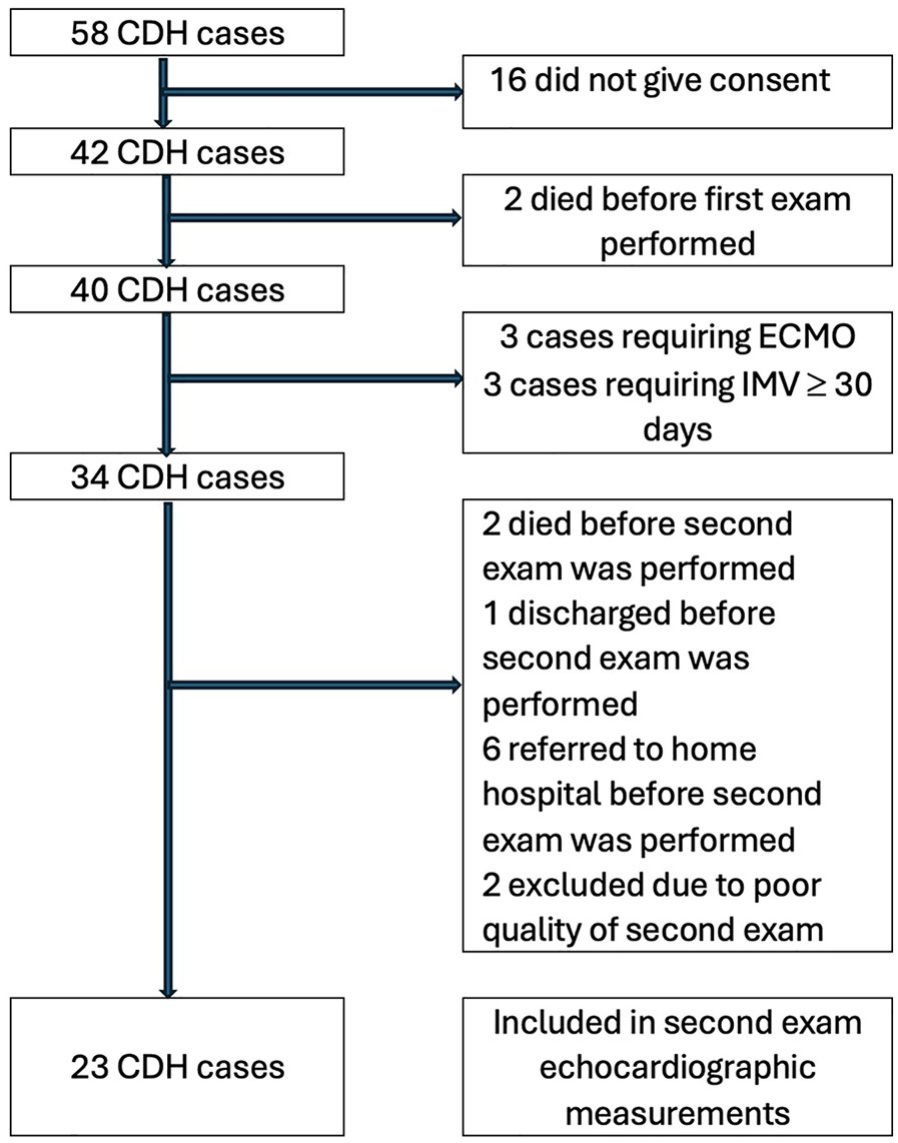
Flow diagram of inclusion and exclusion of the study cohort of children born with congenital diaphragmatic hernia (CDH) and treated at Karolinska University Hospital between January 2021 and March 2024.

Between January 2021 and March 2024, a total of 58 CDH neonatal cases were surgically treated at Karolinska University Hospital. Forty-two cases were included in the study; two died shortly after birth, before an echocardiographic examination was performed. A total of 40 CDH cases had a first study protocol echocardiogram performed between 6 and 24 h of age. The control group (*n* = 40) consisted of newborns from the maternity ward (*n* = 36) and included newborns with anomalies not likely to affect the heart, such as anal atresia and esophageal atresia (*n* = 4). Among the CDH cases, 24 underwent a second echocardiogram at 2–5 weeks of age. One case remained on ECMO at the time of the second examination and was therefore excluded from the analysis of echocardiographic parameters. The CDH subgroup undergoing an examination at 2–5 weeks of age was matched with controls (*n* = 23) from the original control group, who also underwent a second echocardiogram at 2–5 weeks of age. Reasons for exclusion are displayed in Figure 1.

There were no major congenital heart defects in the cohort. Four cases presented with genetic anomalies. One died at 16 h of age, before surgery was performed. Two were excluded from data analysis due to an IMV duration of more than 30 days. The fourth had Wolf Hirshhorn's syndrome, where 50% are born with congenital heart defects. This child was small for gestational age but had a structurally normal heart and an IMV duration of 9 days (37). Demographic characteristics are shown in Table 1.

**Table 1:** Demographic data and CDH characteristics.

Variables	First examination (6–24 h of age)		*p*-value	Second examination (2–5 weeks of age)		*p*-value
	CDH (*n* = 40)	Control (*n* = 40)		CDH (*n* = 24)	Control (*n* = 23)	
Gestational age, weeks mean (range)	38.1 (34.0–42.0)	39.7 (36.0–41.8)	<0.01	38 (34–42)	39.7 (38.0–41.8)	<0.01
Caesarean section, *n* (%)	28 (70)	11 (28)	<0.001	17 (71)	8 (35)	<0.01
Sex, male, *n* (%)	23 (58)	19 (48)	0.502	12 (50)	12 (52)	1.0
Birthweight, kg, mean (range)	3.0 (1.9–4.3)	3.5 (2.2–4.5)	<0.01	3.0 (2.2–4.3)	3.6 (2.7–4.5)	<0.01
Prenatal diagnosis, *n* (%)	29 (73)			18 (75)		
Observed-to-expected lung-to-head ratio (%), mean (range)	47 (21–79)			49 (35–79) (*n* = 16)		
Left-sided, *n* (%)	35 (88)			21 (88)		
Patch repair, *n* (%)	24 (60)			17 (71)		
Non-repair, *n* (%)	1 (3)			0		
CDH study group stage, *n* (%)	39			24		
A	5 (13)			2 (8)		
B	16 (40)			11 (46)		
C	17 (43)			11 (46)		
D	1 (3)			0		
CHD complex, *n* (%)	0			0		
CHD minor, *n* (%)	7 (18)			3 (13)		
Genetic anomaly, *n* (%)	4 (10)			2 (8)		
ECMO, *n* (%)	3 (8)			3 (13)		
Survival (28 days), *n* (%)	38 (93)			23 (96)		
Survival after surgical repair	39 (98)			24 (100)		
LOS (days), mean (range)	20 (1–67)			23 (8–67)		
IMV (days), mean (range)	14 (1–67)			16 (6–67)		
Age at surgical repair (days), mean (range)	4 (2–8)			4 (2–7)		

Values were presented as mean ± standard deviation (SD) for normally distributed variables and as median (interquartile range, IQR) for non-normally distributed variables. Between-group differences were evaluated with Student's *t*-test for normal data and with the Mann–Whitney *U*-test for non-normal data.

### Echocardiography within 6–24 h of birth

There was a significant difference between the CDH cases (*n* = 40) and the controls (*n* = 40) regarding all measured echocardiographic parameters except PAAT and SF. The result remained consistent even when ECMO cases (*n* = 3) were excluded. See Table 2.

**Table 2:** First examination, echocardiographic data.

Variable	CDH (*n* = 40)	Controls (*n* = 40)	*p*-value
LV LS, %	−14.7 (4.6)	−18.8 (2.7)	<0.001
RVFWS, %	−13.9 (4.2)	−22.4 (4.8)	<0.001
LAS, %			
Reservoir	22.8 (9.3)	33.9 (8.2)	<0.001
Conduit	−12.1 (10.0)	−20.5 (5.9)	<0.001
Contractile	−8.7 (5.3)	−13.4 (5.5)	<0.001
RAS, %			
Reservoir	21.8 (6.9)	37.6 (9.2)	<0.001
Conduit	−11.6 (7.2)	−22.5 (7.1)	<0.001
Contractile	−7.7 (6.2)	−15.1 (8.0)	<0.001
TAPSE, mm	5.26 (1.8)	9.4 (1.1)	<0.001
LVED in SD	−2.6 (3)	0.17 (1)	<0.001
SF, %	35 (9)	38 (5)	0.236
PAAT, ms	55 (18.0)	56 (14.3)	0.610
EI	1.8 (0.7)	1.3 (0.2)	<0.001
RV/LV ratio	1.3 (0.64)	0.84 (0.26)	<0.001
TR	35 (87.5)	9 (22.5)	0.026
TR, mmHg	38 (9.6)	17.7 (20)	0.013
PFO	36 (90)	35 (87.5)	1
PFO direction			0.016
Left-right	26 (72)	34 (97)	
Bidirectional	8 (22)	1 (3)	
Right-left	2 (6)	0	
PFO left, (m/s)	0.7 (0.5–0.8)	0.7 (0.6–0.9)	0.64
PDA	34 (85)	22 (55)	0.007
PDA direction			0.001
Left-right	8 (23)	17 (77)	
Bidirectional	22 (65)	4 (18)	
Right-left	4 (12)	1 (5)	
PDA left, (m/s)	0.89 (0.65–1.1)	1.96 (1.62–2.2)	
Septum			<0.001
Round	8 (2)	24 (60)	
Flattened	20 (50)	16 (40)	
D-shaped	12 (30)	0	

Values were presented as mean ± standard deviation (SD) for normally distributed variables and as median (interquartile range, IQR) for non-normally distributed variables. Between-group differences were evaluated with Student's *t*-test for normal data and with the Mann–Whitney *U*-test for non-normal data.

Continuous variables were reported as mean ± standard deviation (SD) when normally distributed and as median (interquartile range, IQR) when non-normal. Student's *t*-test or the Mann–Whitney *U*-test was used for two-group comparisons, as appropriate. For comparisons involving more than two groups, the Kruskal–Wallis test was applied. Categorical variables were analyzed with the *χ*^2^ test or Fisher's exact test.

After exclusion of the most severe cases requiring ECMO support, early deaths, and cases requiring IMV for ≥30 days (*n* = 34), correlations were seen between LV LS and LOS (correlation coefficient −0.344; *p* = 0.047) and between LV LS and duration of IMV (correlation coefficient 0.441; *p* = 0.009). LVED, RVFWS, and LASr did not correlate with LOS or duration of IMV. After adjusting for confounders weight and sex the association between the LV LS and LOS remained statistically significant.

After exclusion of the most severe cases supported with ECMO and cases requiring IMV ≥30 days (*n* = 35), no correlations were found between LASr, LAScd, or LASct and PFO blood flow (left to right velocity gradient). Moreover, there were no correlations between LASr, LAScd, LASct, RASr, RAScd, or RASct and different directions of PFO blood flow.

There were no correlations between RASr, RAScd, or RASct and PDA blood flow (left to right velocity gradient), PAAT, EI, or RV/LV ratio.

No correlations were found between RASr, RAScd, or RASct and different direction of PDA blood flow, nor were any correlations found between RASr, RAScd, or RASct and different septal patterns.

RVFWS correlated with TAPSE (correlation coefficient −0.663; *p* < 0.001), with LASr (correlation coefficient 0.424; *p* = 0.012), with LAScd (correlation coefficient −0.416; *p* = 0.014), and with RASct (correlation coefficient 0.340; *p* = 0.049). RVFWS did not correlate with RASr (*p* = 0.499) or RAScd (*p* = 0.834).

LV LS did not correlate with SF (*p* = 0.342) or LVED SD (*p* = 0.103).

LV LS, LASr and RASr did not correlate with sex, weight or gestational age. RVFWS correlated with sex (correlation coefficient 0.353; *p* = 0.040). After adjusting for confounders PAAT and septal patterns RVFWS did not associate with sex.

In the control group, there were no correlations between RASr, RAScd, or RASct and TAPSE, RV/LV ratio, PAAT, or EI, but there was a correlation between RASr and RVFWS (correlation coefficient −0.317; *p* = 0.046). No correlations were found between RAScd or RASct and RVFWS in the control group. LV LS, LASr, RVFWS and RASr did not correlate with, sex, weight or gestational age.

### Echocardiography at 2–5 weeks of age

The differences in strain and LVED measurements between cases and controls at the first and second examinations, along with the changes in these measurements between examinations, are shown in Table 3.

**Table 3:** Echocardiographic data by subgroups.

Parameter	First examination CDH (*n* = 23)	First examination control (*n* = 23)	*p*-value	Second examination CDH (*n* = 23)	Second examination control (*n* = 23)	*p*-value	Difference between second and first examinations in CDH group (*n* = 23)	Difference between second and first examinations in control group (*n* = 23)	*p*-value
LV LS (%)	−13.8 (4.3)	−19.7 (2.7)	<0.001	−18.1 (4.5)	−21.4 (2.5)	0.004	−4.2 (5.9)	−2.2 (3.0)	0.120
LASr (%)	21.3 (8.6)	33.8 (8.4)	<0.001	30.7 (8.7)	46.4 (9.0)	<0.001	10.6 (10.7)	13.0 (8.8)	0.879
RVFWS (%)	−12.6 (4.11)	−22.6 (2.9)	<0.001	−22.0 (6.6)	−29.1 (4.1)	<0.001	−8.7 (12.8)	−6.6 (4.7)	0.349
RASr (%)	22.7 (7.2)	35.9 (6.5)	<0.001	34.8 (10.2)	44.3 (8.3)	0.001	12.6 (12.3)	8.53 (10.2)	0.218
LVED (SD)	−3.5 (2.0)	−0.13 (0.7)	<0.001	−0.15 (0.97)	0.56 (0.6)	0.005	2.97 (1,5)	0.43 (0.8)	<0.001

Values were presented as mean ± standard deviation (SD) for normally distributed variables and as median (interquartile range, IQR) for non-normally distributed variables. Between-group differences were evaluated with Student's *t*-test for normal data and with the Mann–Whitney *U*-test for non-normal data.

**Figure 2:**
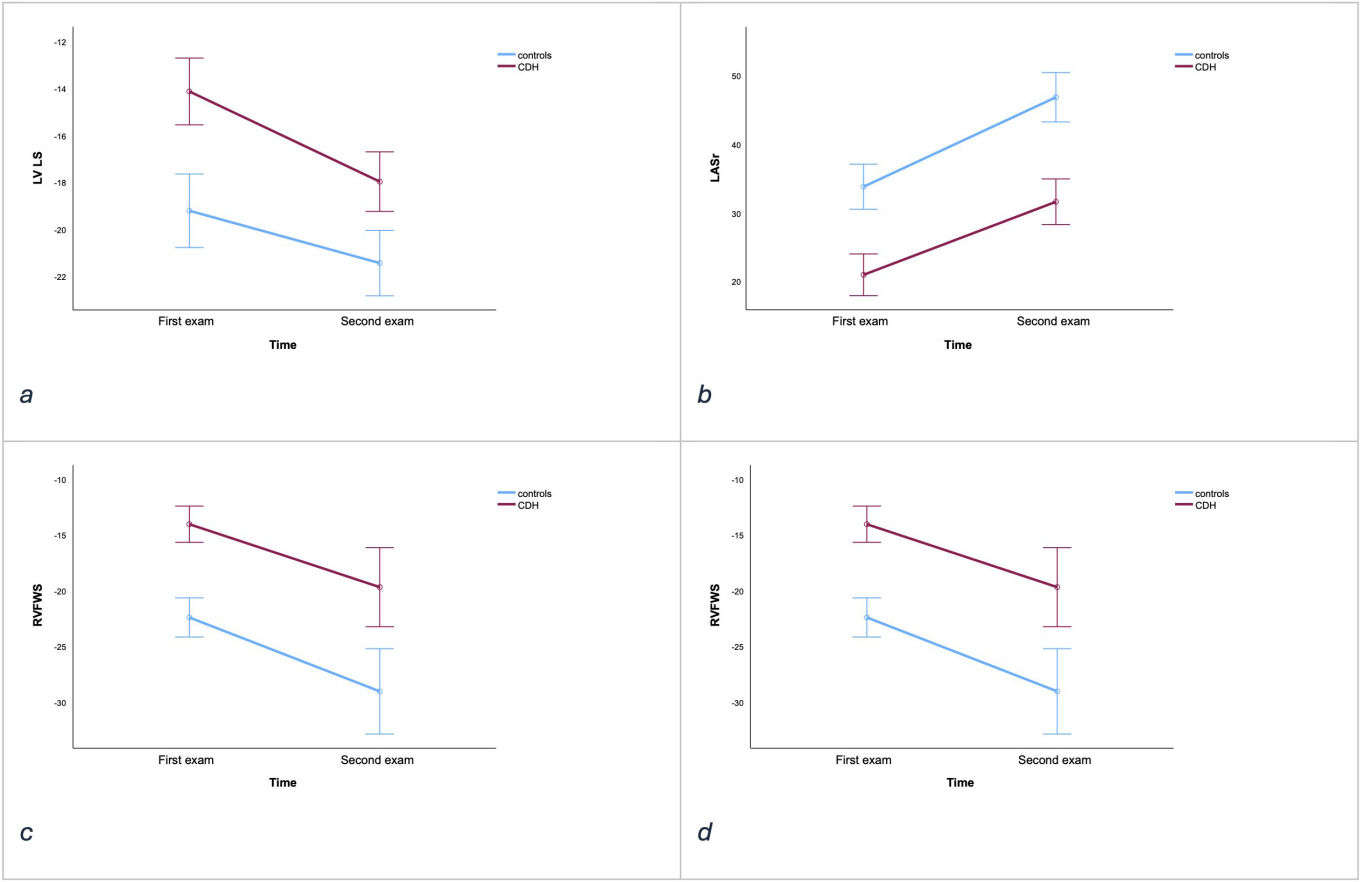
Illustrative figure based on data from Table 3 **(a)** LV LS (%), **(b)** LASr (%), **(c)** RVFWS (%), **(d)** RASr (%), with data from the first and second examinations for CDH cases (red lines) vs. controls (blue lines). *Y* axis; %, *X* axis; time (first examination: 6–24 h of age, second examination 2–5 weeks of age). The lines represent the group-mean values across time points; vertical error bars mark the 95% confidence intervals. In panels **a–d**, cases with CDH differ significantly from controls (see Table 3 for statistics).

Figure 2 visualized that strain improved in both groups over time, yet CDH cases consistently showed reduced strain compared to the controls at each time point (Table 3).

The use of medical treatment is described in Table 4.

**Table 4:** Medical treatment characteristics.

Medical treatment, *n* (%)	CDH first examination (*n* = 40)	CDH second examination (*n* = 24)
Milrinone total,	26 (65)	18 (75)
Milrinone < first 24 h	20 (50)	12 (50)
First examination on milrinone	17 (43)	10 (42)
iN0 total	15 (38)	12 (50)
iNO + milrinone	15 (38)	12 (50)
First examination on iNO	5 (13)	4 (17)
First examination on ECMO	2 (5)	2 (8)
Clinical echo before first examination	13 (33)	8 (33)
Milrinone before clinical echo	1 (3)	1 (4)
Inotrope drug	40 (100)	24 (100)
First examination on noradrenaline	34 (98)	21 (96)
Noradrenaline	40 (100)	24 (100)
>1 inotropic drug	7 (18)	5 (22)
Alprostadil	3 (8)	0 (0)
Sildenafil	11 (28)	9 (38)
Sildenafil oral	8 (20)	7 (29)
Iloprost, inhaled	2 (5)	2 (8)
Epoprostinol	2 (5)	1 (4)
Milrinone + NO + sildenafil	9 (23)	8 (3)
First examination on IMV	40 (100)	24 (100)

After excluding the most severe CDH cases managed on ECMO and cases requiring IMV ≥30 days (*n* = 35), patients with CDH who received milrinone at any point during their PICU stay, including cases given Milrinone within 24 h of birth or at any time during their PICU stay (*n* = 20) were found to have a longer LOS in the PICU and longer duration of IMV compared with CDH patients who did not receive milrinone (*n* = 14) (LOS 20.05 vs. 10.57 days, *p* < 0.001; IMV 11.95 vs. 7.79 days, *p* = 0.007).

Among the CDH patients with early administration of milrinone (<24 h of age) (*n* = 15), there was no significant differences in LOS or duration of IMV compared with the neonates who did not receive early milrinone (*n* = 19) (LOS 17.07 vs. 15.42 days *p* = 0.271; IMV 10.87 vs. 9.74 days, *p* = 0.190).

In the analysis of the CDH subgroup's first examination results, there were no significant differences in the echocardiographic parameters LV LS, RVFWS, LASr RASr, or LVED SD between CDH cases with early administration of milrinone (<24 h of age) (*n* = 12) and those without early milrinone (*n* = 11). Similarly, no significant differences in these parameters were observed between patients who received milrinone at any time during the PICU stay (*n* = 18) and those who did not receive milrinone at all (*n* = 6).

In the analysis of the CDH subgroup's second examination results, there were no significant differences in the echocardiographic parameters LV LS, RVFWS, LASr, RASr, or LVED between patients with early administration of milrinone (<24 h) (*n* = 12) and those without early milrinone (*n* = 11).

In patients treated with milrinone at any point during the PICU stay (*n* = 17), LV LS increased compared with those not treated with milrinone (*n* = 6) (mean LV LS −19.3% vs. −14.8%, *p* = 0.036). There were no significant differences in LASr, RVFWS, RASr, or LVED between patients treated with milrinone at any time during PICU (*n* = 17) and those not treated with milrinone (*n* = 6). However, all parameters demonstrated improvement following milrinone treatment. These results remained when cases managed on ECMO were excluded (*n* = 1).

### Intra- and inter-rater reliability

Table 5 reports the intraclass correlation coefficient (ICC) for echocardiographic strain measurements. ICC ≥ 0.9: Excellent reliability, 0.75 ≤ ICC < 0.90: good reliability, 0.5 ≤ ICC < 0.75 moderate reliability, <0.5 poor reliability (38).

**Table 5:** Reproducibility of echocardiographic measurements assessed by intraclass correlation coefficients (ICC) .

Parameter	Intra-observer ICC (95%CI)	Inter-observer ICC (95%CI)
LV LS	0.90	0.96
LASr	0.90	0.95
LAScd	0.79	0.52
LASct	<0.5	<0.5
RVFWS	0.56	0.98
RASr	0.77	0.80
RAScd	0.56	<0.5
RASct	0.56	<0.5

## Discussion

We demonstrated that newborns with CDH have impaired cardiac function compared with controls. Even though cardiac function improved at 2–5 weeks of age, it remained reduced compared with the control group. One possible explanation is that the group studied at 2–5 weeks of age may have included more severe cases, if milder cases were discharged before the second examination. However, after excluding the case treated with ECMO, no significant differences in IMV duration, patch repair need, or observed-to-expected lung-to-head ratio were observed between this group and those without a second examination, reducing concerns of exclusion bias. This result highlights the need for further research regarding CDH and cardiac function recovery, as remaining impairment might influence long-term cardiac function and cardiac morbidity. Additionally, left ventricular (LV) dimension was measured and no significant difference was found between the CDH group and controls at 2–5 weeks of age. This may be attributed to the relief of mechanical compression following surgical repair and the subsequent increase in blood flow through the left ventricle, which leads to increased ventricular volume and, in turn, an increase in left ventricular dimensions.

There were no correlations found between RASr, RAScd or RASct and conventional echocardiographic parameters indicating pulmonary hypertension in the CHD group. Additionally, no correlations were observed between RASr, RAScd and RVFWS in contrast to findings from previous studies in the adult population (39). There was a weak correlation between RASct and RVFWS in the CDH group. In the Tom Tec version used, the RAS was measured using the LAS algorithm which may have influenced the result. Another confounder could be the complexity of cardiac dysfunction in CDH neonates along with the challenge in obtaining adequate echocardiographic windows for accurate strain measurement. However, nor in the control group were there any correlation between RASr, RAScd or RASct and conventional echocardiographic parameters indicating pulmonary hypertension or potential residual fetal circulation. In the control group there was a weak correlation between RASr and RVFWS as has been demonstrated previous by Hasselberg et al. (39). There were poor to moderate intra- and inter correlation coefficients regarding RAS indicating that the reliability and the reproducibility of RAS in our study population were limited. The findings regarding RAS in this study were contradictory and challenging to interpret. Therefore, the potential effects of using RAS as a marker of pulmonary hypertension and right ventricle diastolic function in the CDH population remain unclear.

In the CDH group, we initially observed a correlation between male sex and reduced RVFWS. However, this association did not persist after adjusting for parameters indicative of pulmonary hypertension, suggesting that sex alone is not independently associated with RVFWS. This lack of correlation is further supported by the control group— a more homogeneous population with fewer confounding factors—where no such association was observed.

Our findings indicate that reduced LV LS in the first echocardiogram correlated with longer stay in PICU and longer duration of IMV. However, we found no association between LVED dimension and either length of PICU stay or duration of IMV, highlighting the ongoing debate about whether LVED size reliably reflects disease severity in this patient population (40). Additionally, we found no correlations between LASr and LOS or IMV, contrary to findings reported in previous studies (13). A possible explanation for this discrepancy might have lain in the personalized treatment strategy we had implemented for neonates with congenital diaphragmatic hernia (CDH) in our unit, which was based on the different hemodynamic phenotypes. In critically ill infants, we routinely performed an early echocardiogram to assess cardiac function. If the echocardiogram revealed left ventricular systolic or diastolic dysfunction or a hypoplastic left ventricle, we withheld inhaled nitric oxide (iNO), even when clinical signs suggested elevated pulmonary pressures. Instead, we were more inclined to initiate milrinone treatment in these cases. As part of our evolving approach, we increasingly focused on the use of left atrial strain as a potentially valuable tool for evaluating cardiac diastolic function in this population. Hence, it is important to note that in 13 CDH cases, an initial clinical echocardiogram had been performed prior to the first study echocardiogram, and in 17 cases, milrinone treatment had been initiated before the study echocardiogram.

Only one case had milrinone treatment started before the early clinical echocardiogram. Thus, performing an echocardiogram within 24 h of age may be crucial for guiding treatment decisions. However, we can only hypothesize that diastolic dysfunction, as assessed through LASr, may respond more effectively to early initiation of milrinone treatment. At the examination at 2–5 weeks, the CDH cases treated with milrinone during the PICU stay presented with improved left ventricle cardiac function, as measured by LV LS, compared with those who did not receive milrinone at all during their PICU stay.

Additionally, our results suggested that initiating milrinone treatment within 24 h of age may be more beneficial in reducing LOS and duration of IMV compared to administration any time during the PICU stay. Notably, there was no difference in LOS or IMV between the early milrinone group and those who did not receive milrinone at all indicating that early administration might be preferable to later treatment. This observation is likely to be multifactorial; later milrinone therapy could be driven by non-cardiac factors, such as infections or respiratory complications, which subsequently impair cardiac function and exacerbate an already vulnerable cardiac function. Moreover, interpretation of milrinone's isolated effect is constrained by the simultaneous administration of other inotropes.

However, early initiation of milrinone treatment may have enhanced cardiac function and thereby improved overall resilience and increased resistance to complications. Compared with in an earlier cohort from Karolinska University Hospital, the use of milrinone and other inotrope drugs had increased (13). Following centralization of CDH surgery in Sweden, there has been a shift in the management of neonates with CDH. Early echocardiograms performed within the 24 h of age and the increased use of inotropes including milrinone is a consequence of more individualized treatment approaches.

### Limitations and strengths

The study had several limitations. First, it included a relatively small cohort. It was an observational study; hence, causative conclusions could not be drawn. However, our findings support the need for a randomized, multicenter study to evaluate long-term cardiac outcomes and the effect of milrinone treatment in the CDH population. The termination of pregnancy rate in Sweden for prenatal CDH is approximately 30% (41). Hence, there may have been a selection bias for less severe cases among the sample of neonates with CDH. Major congenital heart disease and genetic anomalies may have influenced the result as they can be risk factors for LOS, duration of IMV, and mortality (1, 40, 42). Some limitations of the echocardiographic method arise from the influence of factors such as heart rate and the severity of pulmonary hypertension, which can significantly affect the results and make accurate interpretation of the measurements more challenging. Additionally, the transition from fetal to postnatal circulation may affect echocardiographic outcomes, as this transition can vary between cases, even among individuals of the same age.

The study also had strengths. All echocardiograms, on both cases and controls, were performed at between 6 and 24 h of age and at between 2 and 5 weeks by a few experienced technicians in the same pediatric cardiology unit, making comparisons more reliable. All echocardiograms were performed in accordance with a standardized research protocol, ensuring the robustness of the data. One experienced pediatric cardiologist analyzed the data. The data were reviewed separately from the initial care of the study participants and the analyzing pediatric cardiologist was blinded to the outcome of the cases during analysis. The intra- and inter-rater variability regarding strain analyses showed mostly excellent or good results. The strain analysis software used was vendor-independent (TomTec) and all examination results were stored in the same server system. All children, both the CDH group and the control group, were cared for and managed at the same hospital, minimizing the risk of missing medical data. The standardization treatment protocol for children born with CDH at Karolinska University Hospital before, during, and after birth strengthened the robustness of the study findings.

## Conclusion

Cardiac systolic and diastolic function measured as cardiac strain was reduced in newborns with CDH compared with in controls. The cardiac function improved at 2–5 weeks of age but remained impaired compared with in controls. In newborns with CDH, starting milrinone treatment early may be more effective than delaying it or administering it without a specific timing strategy.

## Data Availability

The raw data supporting the conclusions of this article will be made available by the authors, without undue reservation.
