# Supplementary material for: Cardiac function in children with congenital diaphragmatic hernia: cardiac strain at birth and at 2–5 weeks of age
Source: Front Pediatr. 2025 Jul 17;13:1598695. doi: 10.3389/fped.2025.1598695 (PMC12310737; doi:10.3389/fped.2025.1598695)
Supplement: Supplementary file 1 [file Datasheet1.pdf]

Echo protocol CODE-HEART study

Philips, probe 12 or 9, optimize picture
Minimum of 3 consecutive cardiac cycles is required
Electrocardiogram signal with well-defined QRS is required
All M-Mode and Doppler spectral data is to be recorded in “ frozen “ screen format.
Frame rate should be between 90-110 (FR/HR 0,7-0,9)
Sample volume 2mm

Subcostal

1. 2D, situs
2. Color flow doppler of the abdominal aorta
3. 2D, the inferior vena cava
4. 2D and color flow doppler, atrial septum
5. PW spectral doppler atrial septum, PFO
6. 2D (2D and color flow doppler) bicaval view
7. 2D long axis view
8. 2D short axis view

Apical 4

9. 2D(optimize 4 chamber; longest, widest LV cavity area)
10. 2D (optimize 4 chamber LV), FR 90-110
11. 2D image of 4 chamber to optimize visualization of the right ventricle, apical, FR 90-110
12. M mode right ventricle, TAPSE
13. Color flow doppler for the evaluation of MR
14. PW spectral doppler at the trans-mitral flow velocity with sample volume at the mitral valve
15. CW flow doppler for evaluation of MR
16. Color flow doppler for the evaluation of TR
17. CW flow doppler, TR
18. PW spectral doppler at the trans-tricuspid flow velocity with sample volume at the tricuspid valve
19. PW Doppler tissue imaging myocardial velocity mitral annulus(lateral wall)
20. PW Doppler tissue imaging myocardial velocity mitral annulus (septal wall)
21. PW Doppler tissue imaging myocardial velocity tricuspid annulus (lateral wall)
22. PW doppler pulmonary vein

Apical 2

23. 2D, including left atrium (avoid foreshortening; longest widest LV cavity area, FR 90-110

Apical 3

24. 2D, longest widest LV cavity area, FR 90-110

Apical 5

25. 2D
26. Color flow doppler for evaluation of AR

27. PW spectral Doppler of the LVOT /MV (opening and closing click), sweep speed 100mm/sec
28. PW spectral Doppler of the LVOT/MV (opening and closing click , sweep speed 150mm/sec
29. PW spectral Doppler of the aortic valve
30. CW spectral Doppler of the aortic valve

LAX

31. 2D
32. Color flow evaluation of AR
33. Color flow evaluation of MR
34. M-mode of LV for measurements
35. Color flow evaluation of septal wall (VSD?)

SAX

36. 2D LV at papillary muscle level, FR 90-110
37. 2D LV at mitral level, FR 90-110
38. 2D LV at aortic level
39. 2D LV at apex, FR 90-110
40. 2D and color flow coronary arteries.
41. Color flow for evaluation of PR
42. CW flow doppler for evaluation of PR
43. PW spectral Doppler at pulmonary artery level
44. PW spectral Doppler at LPA
45. PW spectral Doppler at RPA
46. 2D and color compare view, RPA, LPA, DA
47. Color flow ductal cut
48. CW spectral Doppler DA
49. PW spectral Doppler DA
50. Color flow pulmonary vein inflow left ventricle (crab view)

Supra sternal view

51. Color flow descending aorta
52. CW spectral Doppler descending aorta
53. PW spectral Doppler descending aorta
